# A Short Version of the Physical Activity Enjoyment Scale: Development and Psychometric Properties

**DOI:** 10.3390/ijerph182111035

**Published:** 2021-10-20

**Authors:** Cheng Chen, Susanne Weyland, Julian Fritsch, Alexander Woll, Claudia Niessner, Alexander Burchartz, Steffen C. E. Schmidt, Darko Jekauc

**Affiliations:** Institute of Sports and Sports Science, Karlsruhe Institute of Technology (KIT), Engler-Bunte-Ring 15, 76131 Karlsruhe, Germany; susanne.weyland@kit.edu (S.W.); julian.fritsch@kit.edu (J.F.); alexander.woll@kit.edu (A.W.); Claudia.Niessner@kit.edu (C.N.); alexander.burchartz@kit.edu (A.B.); steffen.schmidt@kit.edu (S.C.E.S.); darko.jekauc@kit.edu (D.J.)

**Keywords:** content analysis, PACES, physical activity, reliability, validity

## Abstract

Objective: The purposes of this paper were to (a) develop a new short, theory-driven, version of the physical activity enjoyment scale (PACES-S) using content analysis; and (b) subsequently to measure the psychometric properties (construct validity, internal consistency, test–retest reliability, and concurrent validity) of the PACES-S for adolescents. Methods: Six experts used a four-point Likert scale to assess the content validity of each of the 16 items of the physical activity enjoyment scale according to a provided definition of physical activity enjoyment. Based on the results, exploratory factor analysis was used to analyze survey data from a longitudinal study of 182 individuals (Measure 1 of Study 1: 15.75 ± 3.39 yrs; 56.6% boys, 43.4% girls), and confirmatory factor analysis (Measure 2 of Study 1: 15.69 ± 3.44 yrs; 56.3% boys, 43.7% girls) was used to analyze the survey data from a cross-sectional study of 3219 individuals (Study 2; 15.99 ± 3.10 yrs; 47.8% boys, 52.2% girls) to assess the construct validity of the new measure. To assess the reliability, test–retest reliability was assessed in Study 1 and internal consistency in Study 1 and 2. For the concurrent validity, correlations with self-reported and device-based physical activity behavior were assessed in both studies. Results: Four out of sixteen items were selected for PACES-S. Exploratory factor analysis and confirmatory factor analyses identified and supported its factorial validity (χ^2^ = 53.62, df = 2, *p* < 0.001; RMSEA = 0.073; CFI = 0.99; RFI = 0.96; NFI = 0.99; TLI = 0.96; IFI = 0.99). Results showed good test–retest reliability (r = 0.76) and internal consistency (*a* = 0.82 to 0.88). Regarding concurrent validity, the results showed positive correlations with a physical activity questionnaire (Study 1: r = 0.36), with a physical activity diary (Study 1: r = 0.44), and with accelerometer-recorded data (Study 1: r = 0.32; Study 2: r = 0.21). Conclusions: The results indicate that PACES-S is a reliable and valid instrument that may be particularly useful to measure physical activity enjoyment in large-scale studies. It shows comparable measurement properties as the long version of PACES.

## 1. Introduction

The scientific evidence allows the conclusion that physical activity (PA) during adolescence contributes to developing a healthier lifestyle in later life, reducing the prevalence of non-communicable diseases and improving psychological well-being [1,2,3,4]. According to the WHO recommendations on the health benefits of PA, adolescents should accumulate at least 60 min of moderate to vigorous PA per day [5]. However, only a minority of adolescents report engaging in PA at a level compatible with the health guidelines [6,7,8]. Moreover, while many adolescents start PA programs to improve their health and lose weight, the rate of dropouts is high [9]. Specifically, regarding the maintenance of PA, researchers emphasize the role of affective processes [10,11,12]. Notably, there is a large volume of studies describing the critical role of enjoyment in PA [13,14,15,16,17,18,19]. Despite extensive research demonstrating the importance of PA enjoyment, to date, however, there has been little consensus on what PA enjoyment actually is [20].

In general, enjoyment can be regarded as an emotion. There has been a long debate about how emotion might be defined. One study has collected a long list of definitions of emotion, none of which have been able to gain general acceptance [21]. One well-known study stated that a distinction should be made between an automatic affect and a full-blown emotion [22]. While automatic affect represents a simple and rapid appraisal that something is good or bad, positive or negative, emotions are more deliberate, slow, and involve cognitive processes. Although there is currently no universal definition of emotion, most scientists agree that emotions always represent a valenced state of relatively short duration and are related to an object, person, or activity [23]. Based on the component process model [24], five emotion components can be distinguished: cognitive appraisal, physiological responses, action tendencies, motor expressions, and feelings (also called subjective experience). The components are recursively influenced by appraisal processes, contributing to their consistency and synchronization [24,25]. All these changes are then integrated and centrally represented as feelings [25], which are then further categorized and labeled as emotional terms (e.g., enjoyment). That is, the feeling component is considered the most central component of emotion, which differentiates it from other psychological states [26]. Based on these theoretical considerations, we define PA enjoyment as a positively valenced emotion directed towards PA associated with feelings such as pleasure, joy, and fun [27].

In measuring PA enjoyment, the physical activity enjoyment scale (PACES) is the most prominent instrument. While the original version was developed by Kendzierski and DeCarlo [28], several alternative forms have been developed (see Table 1 for a comparison). In detail, the original unidimensional 18-item PACES [28] was validated for its validity and reliability (α = 0.93) in college students (aged between 18–24 years). However, a factor analysis of the PACES in the youth sports population(aged between 10–17 years) showed that the scale was not unidimensional [29]. After evaluating by a focus group, two items were removed [30], one of which (“I was very absorbed in the activity”) was removed because it was considered to be irrelevant to PA enjoyment, the other (“It is very invigorating”) was removed because it was considered redundant. However, the study also reported that the 16-item PACES fitted a unidimensional model with methodologic effects behind positively worded items [30]. Given this, Dishman et al. [31] eliminated the positively worded items reducing the scale items to seven and identified sufficient construct validity of the seven-item scale in a sample of US adolescents. However, one study argued that many scale shortening studies do not start from a conceptual point of view but place excessive credit on statistical techniques [32]. Then PA enjoyment was defined as a positive response to the movement experience or an optimal psychological state that leads to performing PA [33]. Raedeke noted that the 18-item PACES appears to tap not only into PA enjoyment (i.e., PA enjoyment reflects feelings about exercise and is a psychological state directly connected to an eliciting stimulus—the exercise experience) itself but also the potential antecedents and consequences of PA enjoyment. Therefore, content analysis with four experts was implemented to shorten the 18-item PACES, and ten items were removed because they were considered not to be the generalized state of enjoying PA or the experience itself. However, the inclusion of an item, “I was very absorbed in the activity,” conflicts with Motl et al.’s [30] results (“I was very absorbed in the activity” was removed because the content was considered not relevant to enjoyment). Furthermore, Raedeke [33] only reported the item-total correlation and did not attempt to identify other psychometric properties (e.g., construct validity, test–retest reliability, and concurrent validity). In summary, various forms of PACES have been developed for which different limitations have been identified (e.g., the inadequate conceptualization of the PA enjoyment, the methodological effect of positively and negatively worded items). It can be assumed that the methodologic effect is based on an inadequate conceptualization of the construct enjoyment and that the items of PACES might contain contents of further similar constructs [27]. To address these limitations, we argued that it might be helpful to use the definition mentioned above of PA enjoyment as a starting point to develop a new, shortened scale based on the long versions of PACES.

The purpose of this article was to provide a new form of PACES, using those items that are in line with the definition of PA enjoyment as “PA enjoyment as a positively valenced emotion directed toward the PA associated with feelings such as pleasure, joy, and fun.” This implies a reduction of items since we are only interested in those items that truly reflect the subjective experience of PA enjoyment. We believe it could be further beneficial because it can reduce the burden on participants and be more easily used in large-scale studies [34,35]. Hence, the first aim of this paper was to use content analysis to preliminary develop a new short scale. Based on the results of this procedure, the second aim was to measure the psychometric properties of the shortened scale. These include (a) construct validity, (b) internal consistency, (c) test–retest reliability, and (d) concurrent validity. To achieve these aims, first, experts were asked to evaluate the content validity of the individual items of PACES based on the definition of the provided PA enjoyment. Subsequently, the data collected in two studies [36,37] (the original authors and project director were contacted to obtain the original PA and PA enjoyment measurement data) were used to determine the psychometric properties of the new PACES.

## 2. Phase 1: Content Analysis

### 2.1. Method

According to Lynn [38], at least five experts were required to provide sufficient control over the chance agreement. Therefore, six experts were selected. Four of these six experts held doctoral degrees in sports science, three of which hold professorships in sports psychology (based in Germany or Switzerland), and one held a research fellowship in sports management in Germany. The other two experts were a Ph.D. student in sports psychology and a master student in sports science in Germany, respectively. To determine the content validity index, the definition of PA enjoyment (i.e., PA enjoyment is a positively valenced emotion directed toward PA associated with feelings such as pleasure, joy, and fun) was provided based on the component process model [24]. Experts were explicitly asked to consider whether negatively worded items (e.g., it is not fun at all) could also measure PA enjoyment. A modified four-point Likert scale (1 = “does not match the definition”; 2 = “matches the definition somewhat well”; 3 = “matches the definition quite well”; 4 = “matches the definition very well”) [39] was used to assess the content validity of each of the 16 items [30,40]. By calculating the results of the experts’ evaluation, a new short version of the German PACES would then be preliminary developed, subsequently referred to as PACES-S.

### 2.2. Data Analysis: Content Validity (Item Selection)

The statistical analyses of content validity were performed in Microsoft Excel [41] using the formulas below.

A four-point Likert scale, clearly labeled with the definition of PA enjoyment and the content of each item, was sent to each expert separately. They were invited to rate the relevance of each item according to the definition of PA enjoyment independently. Based on the experts’ evaluation results, ratings of 1 or 2 for each item were considered unacceptable, and 3 or 4 were considered acceptable [38]. Two types of content validity indices were used to assess and delete items: (a) item-level content validity index (I-CVI; i.e., the number of experts assigned Grade 3 or 4, divided by the total number of experts) [39]; (b) the scale-level content validity index calculated by the average method (S-CVI/ Ave; i.e., the average proportion of items assigned either Grade 3 or 4 across judges) [42].

When N experts evaluated one item, of which n_1_ experts assigned it a rating of 1 or 2 and n_2_ assigned it a rating of 3 or 4 (N = n_1_ + n_2_), the I-CVI could be computed as:

$$I-\mathrm{CVI}=\frac{n_{2}}{N}$$

However, the results derived from the above equations ignored the chance agreement. Therefore, Polit and Beck [42] and Wynd et al. [43] advocated adjusting I-CVI calculation and using k* to denote the adjusted I-CVI results. To compute k*, the probability of chance agreement (P_c_) was calculated first. The formula was as follows:

$$P_{c}=\left[\frac{N!}{n_{2}!\left(N-n_{2}\right)!}\right].5^{N}$$

Next, k* was computed using the I-CVI and P_c_:

$$k^{*}=\frac{I-\mathrm{CVI}-P_{C}}{1-P_{c}}$$

Then, if a scale had n items and the data value was I-CVI_i_ (i = 1, 2, …, n), then we had:

$$S-\mathrm{CVI}/\mathrm{Ave}=\frac{1}{n}\overset{n}{\underset{i=1}{\sum }}\left(I-{\mathrm{CVI}}_{i}\right)$$

Finally, k* and S-CVI/Ave were employed to evaluate the acceptability of the scale in item level and overall level, respectively. With six experts, the evaluation criteria for k* were as follows: below 0.40 indicated “poor” validity, 0.40 to 0.59 indicated “fair” validity, 0.60 to 0.74 indicated “good” validity, and greater than 0.74 represented “excellent” validity [44,45]. Polit and Beck [42] recommended that a scale should be composed of items with k* of 0.74 or higher and S-CVI/Ave of 0.90 or higher.

### 2.3. Result

Based on the content validity evaluated by six experts, four out of sixteen items have been selected. All these four items showed k* higher than 0.74, and the S-CVI/Ave of the PACES-S was 0.96 (see Table 2). The items included in the PACES-S were: “I enjoy it”, “I find it pleasurable”, “It is very pleasant”, and “It feels good”.

## 3. Phase 2: Psychometric Properties

### 3.1. Method

The data of two cohort studies [36,37] were used to determine internal consistency, test–retest reliability, construct validity, and concurrent validity of the PACES-S developed in Phase 1. The subjects’ PA enjoyment and PA data were measured in Study 1 (Measure 1, Measure 2) and Study 2, respectively.

#### 3.1.1. Study 1

1. Participants

A total of 182 students (male, *n* = 103, female, *n* = 79) aged between 11–17 years were recruited for this study. All students came from a comprehensive secondary school in a German city, with all three types of the traditional German tripartite secondary school system: Hauptschule, Realschule, and Gymnasium. After the teachers had agreed, and according to the Helsinki Declaration, informed written consent was obtained from the participants and their parents or guardians before entering the study [46]. The study was approved by the ethics committee of the Charité Universitätsmedizin Berlin. Detailed information on the data collection techniques and quality of the sample are presented elsewhere [36].

2. Procedure

Participants provided their personal information (e.g., age, gender, school type). They also completed the MoMo physical activity questionnaire (MoMo-PAQ) and the PACES-S twice (Measure 1, Measure 2; Measure 1 and Measure 2 correspond to the PACES-S administered before and after seven days, respectively) at school, with a 7-day interval between the completions. During these seven days, participants wore accelerometers and completed Previous Day Physical Activity Recall (PDPAR; [47]) daily. This study was performed between April and July 2009.

3. Measurement

*Physical activity enjoyment.* The 16-item PACES was used in this study [30,40]. However, based on the results of the content analysis described above, we only included the four items of PACES-S (i.e., Item 1: I enjoy it; Item 2: I find it pleasurable; Item 3: It is very pleasant; Item 4: It feels good) [40]. The items were answered using a five-point Likert scale ranging from 1 = “strongly disagree” to 5 = “strongly agree”.

*PA questionnaire.* Habitual PA was measured by MoMo-PAQ [36]. This questionnaire contained 28 items and measured PA in four distinct settings: daily PA, school PA, PA in and outside organized sports clubs. For each setting, the frequency, duration, intensity, and types of PA were measured. MoMo-PAQ has been shown to be a validated instrument with acceptable reliability (test–retest reliability = 0.68) and significant correlations with accelerometer-recorded data (r = 0.29) [36].

*PA diary.* The PDPAR [47] is a self-reporting and time-based recall instrument designed to capture adolescents’ previous day’s PA. In the present study, certain *hours* of a day were divided into one-hour metric blocks. Participants were instructed to note their specific activities (38 activities were listed for participants to select from, which could be grouped into six main clusters: eating, sleep/bathing, transportation, work/school, spare time, PA) and the intensity of activity for each time block (light, moderate, vigorous, very vigorous). Finally, the metabolic equivalent (MET) levels were computed to determine each participant’s PA. The instrument has proven to be valid and reliable in measuring PA [47,48].

*Accelerometer.* The Actigraph GT1M accelerometer (Pensacola, FL, USA) was also used to measure PA. It is a two-axis accelerometer with a solid-state sensor and micro-electro-mechanical system with a dynamic range of 0.05–2.5 G and frequency range of 0.25–2.5 Hz. The filtered acceleration signal was digitized, rectified, integrated (calculating the ‘activity count’), stored, and reset at user-specified intervals (10 s for the present study). Ultimately, we evaluated the participants’ daily PA based on the duration and intensity of PA (light < 3 METs, moderate 3–6 METs, vigorous 6–9 METs, very vigorous > 9 METs) measured and calculated by accelerometers. In particular, the duration of moderate, vigorous, and extreme vigorous PA per day was combined into a single variable as “accelerometer-recorded MVPA”. The accelerometers were worn around the participants’ waists via elastic waistbands. Participants were requested to wear the devices for seven consecutive days of waking hours (except for swimming and bathing). Measuring PA with the Actigraph GT1M has been proven valid and reliable for adolescents [49,50]. Eligible accelerometer data should meet the criteria that: (1) participants wore the accelerometer for at least 10 h per day over a minimum of 5 days, and (2) non-wearing was defined as at least 60 consecutive minutes of zero activity intensity (1–2 min of counts between 0 and 100 were allowed).

#### 3.1.2. Study 2

To replicate the reliability and validity analyses of the PACES-S in Study 1, psychometric properties of the measure were also assessed using data from Study 2 [37].

1. Participants

The German Health Interview and Examination Survey for Children and Adolescents (KiGGS) is part of the Federal Health Monitoring System conducted by the Robert Koch Institute (RKI) and consists of regularly conducted nationwide surveys among children, adolescents, and young adults aged 0 to 29 years and living in Germany. KiGGS Wave 2 was conducted between 2014 and 2017. The Motorik-Modul Study (MoMo) is a submodule of the KiGGS study and aims to assess physical fitness, PA, as well as determinants of PA in children and adolescents [51]. The whole study sample was drawn from the German resident population aged 4 to 17 years (only subjects aged between 11 and 17 years were selected for this study) using a two-stage cluster sampling approach. Informed consent to participate in the study was obtained from the participants and their parents or guardians. In addition, participants from the baseline study (2003–2006) and Wave 1 (2009–2012) were reinvited. A detailed description of the study design and sampling procedure can be found elsewhere [37,52,53]. KiGGS and MoMo provide nationally representative data of PA and sedentary behavior of children, adolescents, and young adults living in Germany [52]. A favorable vote of the ethics committee of Karlsruhe Institute of Technology of 23 September 2014, is available for the study. A total of 3219 participants (male, *n* = 1538, female, *n* = 1681) aged between 11–17 years were recruited for this study.

2. Procedure

Participants provided their personal information (e.g., age, gender, school type) and completed the PACES-S after physical fitness tests [54]. After completing the scales, participants were assigned to wear accelerometers for eight days to record their PA data (data measured on the first day were discarded. This study was performed between 2014 and 2017.

3. Measurement

*Enjoyment.* Enjoyment was measured using the PACES-S described in Study 1.

*Accelerometer.* PA was measured using the Actigraph GT3X, the successor accelerometer model described in Study 1. The technical and methodological details of the accelerometer measurement of Study 2 can be found elsewhere [52]. In short, placement of the device was on the hip, sampling frequency was 30 Hz, the same filter as in Study 1 was used, epoch lengths was 1 s with the possibility to convert into 5 s, 10 s, 15 s, 30 s, and 60 s, non-wear time definition was the algorithm by Choi et al. [52], and the valid datasets needed eight hours of recordings on four weekdays and one further weekend day when wearing the device for seven days. Sedentary and physical activity intensity classification used algorithms by Evenson et al. [55] and Romanzini et al. [56]. In addition, the number of days that each participant met the WHO physical activity recommendation level (i.e., Daily MVPA greater than 60 min; [5] over seven days was combined into a new variable, “PA compliance days”.

### 3.2. Data Analysis (Study 1 and 2)

For psychometric properties, we evaluated the internal consistency, test–retest reliability, construct, and concurrent validity of the PACES-S.

*Internal consistency*. The PACES-S data from Study 1 (Measure 1, Measure 2) and Study 2 were used to analyze internal consistency in SPSS 25 [57]. The internal consistency was assessed by examining Cronbach’s alpha coefficient [58]. An acceptable alpha value would be in the range of 0.70 to 0.90 [59,60].

*Test–retest reliability.* The PACES-S scores measured twice a week apart in Study 1 were used to calculate Pearson correlation coefficients in SPSS 25. A 5% cut-off was taken for significance, whereby a value greater than 0.70 was deemed to be acceptable [61].

*Construct validity.* Factor analyses were conducted to assess construct validity based on the results of the PACES-S from Study 1 (Measure 2) and Study 2. Data from Study 1 (Measure 2) were used for an exploratory factor analysis (EFA) to explore the underlying structure of the PACES-S in SPSS 25 [62]. Then, data from Study 2 were used for a confirmatory factor analysis (CFA) to validate the identified factor structure in AMOS 25 [63,64]. Firstly, the factors were extracted in EFA using the principal component method with varimax rotation. The Kaiser–Meyer–Olkin (KMO) measure of sampling adequacy and Bartlett’s test of sphericity were employed to test the appropriateness of the factor analysis [65]. Missing data ranged between 0.5–2.7% for the PACES items. Further, the specific evaluation criteria were as follows: (1) the factor loading of an item was not less than 0.6 [66]; (2) the number of factors was determined using a scree plot [67] and the following criteria: eigenvalue greater than 1 [68,69], an individual factor accounting for no less than 10% of the total variance, and a composite of the extracted factors accounting for no less than 70% of the total variance [70]. Secondly, CFA was used to validate the structure obtained in EFA using full-information maximum likelihood estimation. This method yields less biased estimates than classical missing data procedures, such as list-/pairwise deletion or means imputation [71]. Missing data ranged between 1.9–2.5% for the PACES items. Given the high sensitivity of Chi-square statistics in large samples [72], the following fit indices and criteria were used to examine the goodness of fit of the model (it was considered good if the following criteria were satisfied): root mean square error of approximation (RMSEA) between 0 and 0.08 [73]; comparative fit index (CFI), normed fit index (NFI), relative fit index (RFI) and Tucker-Lewis Index (TLI) between 0.95 and 1, and incremental fit index (IFI) over 0.90 [74,75,76].

*Concurrent validity.* The concurrent validities for PACES-S were derived by computing Pearson correlation coefficients between PACES-S scores (Measure 2) and criterion scores for MoMo-PAQ [36], PDPAR [47], and accelerometer (“accelerometer-recorded MVPA”) in Study 1. Simultaneously, the correlations between results on accelerometers (“accelerometer-recorded MVPA” and “PA compliance days”) and PACES-S provided the estimate of concurrent validity in Study 2. A 5% cut-off was used for significance, with four levels of interpretation for correlation-based effect sizes: very small (r < 0.1), small (0.10 ≤ r ≤ 0.30), moderate (0.30 ≤ r < 0.50), large (0.50 ≤ r) [77].

### 3.3. Results

#### 3.3.1. Study 1

1. Descriptive Statistics

Of the 182 participants, 103 (56.6%) were males, and 79 (43.4%) were females. Regarding age distribution, 111 (61.0%) were between 11 and 13 years old, 71 (39.0%) were between 14 and 17 years old. Different types of schools accounted for the following percentages of participants: Hauptschule (14.8%), Realschule (30.8%), and Gymnasium (54.4%). As can be seen in Table 3, the overall data of 174 PACES-S data and participants were valid (missing or invalid data: PACES-S (time 1), *n* = 8, PACES-S (time 2), *n* = 8, accelerometer, *n* = 2; PA questionnaire, *n* = 0, PA diary, *n* =0). Concerning males only, 100 (97.1%, missing or invalid data, *n* = 3) and 98 (95.1%, missing or invalid data, *n* = 5) participants’ PACES-S data were valid for time 1 and time 2, respectively. All (*n* = 103, 100%) male participants’ accelerometer, PA questionnaire, and diary data were valid, 101 (98.1%) male participants’ accelerometer data were valid (missing or invalid data, *n* = 2). For females only, 74 (93.7%, missing or invalid data, *n* = 5) and 76 (96.2%, missing or invalid data, *n* = 3) participants’ PACES-S data were valid for time 1 and time 2, respectively. All (*n* = 79, 100%) female participants’ accelerometer, PA questionnaire, and diary data were valid.

2. Internal Consistency

As seen in Table 3, for Measure 1 of Study 1, the overall Cronbach’s alpha for the PACES-S was 0.83, for male participants, the Cronbach’s alpha for the PACES-S was 0.82, and the Cronbach’s alpha for the PACES-S for female participants was 0.85.

For Measure 2 of Study 1, the overall Cronbach’s alpha for the PACES-S was 0.86, for male participants, the Cronbach’s alpha for the PACES-S was 0.87, and the Cronbach’s alpha for the PACES-S for female participants was 0.83.

3. Test–Retest Reliability

The stability coefficient of the PACES-S for a one-week interval was found to be significant and sufficiently high (r = 0.76, t = 15.14, df = 165, *p* < 0.01).

4. Construct Validity

In EFA, the results of Study 1 (Measure 2) showed KMO=0.80, Bartlett’s test of sphericity χ^2^ = 313.18, df = 6, *p* < 0.001, indicating that the data were suitable for the factor analysis. Following the principle of eigenvalues greater than 1 and the scree plot to assess the results of the principal component analysis, we identified one factor (eigenvalue = 2.82), which explained 70.38% of the total variance. The factor loadings for the items ranged from 0.79 to 0.86 (see Table 4).

5. Concurrent Validity

We found a moderate correlation between scores on the PACES-S and the MoMo-PAQ, r = 0.36, t = 4.98, df = 173, *p* < 0.001; a moderate correlation between the PACES total score and PDPAR (MVPA minutes) results, r = 0.44, t = 6.34, df = 173, *p* < 0.001; and a moderate correlation between the PACES-S scores and the accelerometer criterion (accelerometer-recorded MVPA), r = 0.32, t = 3.48, df = 109, *p* < 0.001.

#### 3.3.2. Study 2

1. Descriptive Statistics

Of the 3219 participants, 1538 (47.8%) were males, and 1681 (52.2%) were females. In terms of age distribution, 1343 (41.7%) were between 11 and 13 years old, and 1876 (58.3%) were between 14 and 17 years old. Different types of schools accounted for the following percentages of participants: Grundschule (1.8%), Hauptschule (3.5%), Realschule (22.2%), Gymnasium (50.7%), Gesamtschule (9.1%), Förderschule (0.7%), and other types of schools or missing data (11.87%). As shown in Table 5, the overall data of 3118 PACES-S data were valid (missing or invalid data: PACES-S, *n* = 101, accelerometer, *n* = 1318). Concerning males only, 1493 (97.1%) participants’ PACES-S data were valid (45 missing or invalid data), 885 (57.5%) participants’ accelerometer data were valid (653 missing or invalid data). For females only, 1625 (96.9%) participants’ PACES-S data were valid (56 missing or invalid data), 1016 (60.4%) participants’ accelerometer data were valid (665 missing or invalid data).

2. Internal Consistency

As seen in Table 5, for Study 2, the overall Cronbach’s alpha for the PACES-S was 0.87, for male participants, the Cronbach’s alpha for the PACES-S was 0.88, and the Cronbach’s alpha for the PACES-S for female participants was 0.87.

3. Test–Retest Reliability

The stability coefficient of the PACES-S for a one-week interval was found to be significant and sufficiently high (r = 0.76, t = 15.14, df = 165, *p* < 0.01).

4. Construct Validity

We further used data from Study 2 to test the one-factor model (identified through EFA in Study 1) fit of the PACES-S in AMOS and the overall results indicated a good model fit (χ^2^ = 53.62, df = 2, *p* < 0.001; RMSEA = 0.073; CFI = 0.99; RFI = 0.96; NFI = 0.99; TLI = 0.96; IFI = 0.99).

5. Concurrent Validity

We found a small correlation between scores of PACES-S and the accelerometer-recorded MVPA, r (1840) = 0.21, t = 9.19, *p* < 0.001; and a small correlation between the PACES-S scores and the accelerometer criterion PA compliance days, r (1840) = 0.20, t = 8.78, *p* < 0.001.

## 4. Discussion

This study aimed to develop a new short, theory-based version of PACES, as there was no reliable version for German adolescents. To this end, first content validity was used to select items that matched the definition of PA enjoyment” PA enjoyment as a positively valenced emotion directed toward the PA associated with feelings such as pleasure, joy, and fun.” Subsequently, psychometric properties of the new measures were assessed (i.e., construct validity, internal consistency, test–retest reliability, concurrent validity). Based on the internal consistency and test–retest reliability, the results indicate the good reliability of the new measure. Moreover, both exploratory and confirmatory factor analyses showed a good construct validity of the measure. Finally, regarding the concurrent validity, the results showed that PACES-S positively correlated with self-reported and device-based measures of physical activity.

### 4.1. Item Selection for Short-Version Scale (Content Validity)

Previous studies have pointed to the inappropriateness of the unidimensional factor and redundant items in the original 18-item PACES [28,30] and the methodological effect of negatively worded items in the 16-item PACES [30,40]. Thus, Dishman et al. [15] and Raedeke [33] shortened the scales and obtained a seven-item PACES and an eight-item PACES, respectively. However, the psychometric properties were not adequately validated for the 7-items PACES [15,78], and the theoretical conceptualization was missing for the 8-items PACES [33].

To solve the issue of inadequate conceptualization, we conceptualized PA enjoyment based on the Component Process Model [24] and adopted the methodology of Davis and Polit and Beck [42] to select items. The analytical results found that only 4 of the 16 items achieved the benchmark value for retention (k* ≥ 0.74), and the S-CVI/Ave for the shortened scale was 0.96, indicating that the PACES-S had excellent item-level and scale-level content validity indices. Although the experts were explicitly asked to consider that some items are negatively worded with a higher number indicating a low level of PA enjoyment, the procedure resulted in only positively worded items. Including only positively worded items showed similarity to Raedeke’s [33] experts’ assessment.

### 4.2. Internal Consistency and Test–Retest Reliability

The results indicated good reliability with Cronbach’s alpha ranging from 0.82 to 0.88 and test–retest reliability of 0.76. These values were comparable to studies measuring the psychometric properties of other forms of PACES [30,40]. The values were a bit lower than Kendzierski and DeCarlo (α = 0.96) [28]. However, considering that Kendzierski and DeCarlo’s alpha value is greater than 0.9, as pointed out by Tavakol and Dennick [60], this might imply the presence of redundant items in the scale. Compared to the results of Jekauc et al. [36], the internal consistency is similar to the long version of the PACES.

### 4.3. Construct Validity

The exploratory factor analysis showed that all items were on a single factor. The CFA was then conducted to verify the one-factor solution. Overall, the fit indices indicated that the one-factor model did represent an acceptable fit. Thus, it represented that PACES-S was not suffered from method effects similar to the long version of PACES [30].

### 4.4. Concurrent Validity

The PACES-S presented adequate concurrent validities with total MoMo-PAQ (r = 0.36), PDPAR (r = 0.44), accelerometer-recorded MVPA (Study 1: r = 0.32; Study 2: r = 0.21), and accelerometer-recorded PA compliance days (r = 0.20). Taken together, the PACES-S displayed small to moderate significant correlations with both self-reported PA and accelerometer-measured PA. Similarly, Jekauc et al. [40] measured the predictive validity of the original German version of the 16-item PACES and showed that the scale significantly correlated with the MoMo-PAQ, PDPAR, and accelerometer-recorded MVPA results in German adolescents. Besides, the acceptable concurrent validity between PACES (16 items) and self-reported PA was also in line with the result (r = 0.16, *p* < 0.01) by Moore et al. [79] concerning American children and adolescents. The results of this investigation were also consistent with other studies [80,81] that identified PA enjoyment as an important motivating factor for adolescent participants in PA.

### 4.5. Strengths and Limitations

Based on the component process model [24], the study provided a theory-based definition of PA enjoyment to develop a new version of PACES. This study utilized a reasonably large sample (Study 2) and a smaller sample (Study 1) to investigate the psychometric properties of the PACES-S. This procedure resulted in a new shortened version of PACES that may be particularly useful to reduce the burden of participants in large-scale studies, where a wide range of variables are measured. However, there were still some limitations. First, we did not measure PA enjoyment by more objective indicators (e.g., face expression). However, it is crucial to consider that the objective measure of discrete emotions is highly debated within the scientific community [82]. Moreover, the current results are based on studies with German-speaking participants. Therefore, future studies should try to replicate the findings in other languages. Besides, the research did not include children under 11 years old. We presume that children could benefit from this short version with graphical illustration. Further research could be refined and implemented among them. Finally, the technical development is a normal process, but we think that it should be mentioned in any case that Study 2 used the newer model of the accelerometer with three-dimensional accelerometer acquisition instead of one dimension in Study 1. On the other hand, Kaminsky and Ozemek [83] compared both models used in this investigation and concluded that the data are comparable with each other, whereby the comparability with our data should remain given as well.

## 5. Conclusions

In conclusion, the four-item PACES-S offered a short and economical measure of PA enjoyment based on a comprehensive definition derived from the component process model. The investigations of the psychometric properties indicated good reliability and validity of the measure, which were comparable to the reliability and validity of the 16-item version of the PACES. The two studies showed that the method effect underlying the 16-item version of PACES could be eliminated. We hope that the use of PACES-S will contribute to a better understanding of the role of PA enjoyment in PA promotion and maintenance research.

## Figures and Tables

**Table 1:** Characteristics of different versions of PACES and reasons for item deletions.

Author (Year)	Kendzierski and DeCarlo [28]; Crocker et al. [29]	Raedeke [33]	Motl et al. [30]	Dishman et al. [31]
Version	18-item PACES	8-item PACES	16-item PACES	7-item PACES
Factor	1 factor	1 factor	1 factor	1 factor
Point	7 points	7 points	5 points	5 points
Subject	College students/ youth sports population	Young female adults/ old adults	Adolescents	Children
Items				
Item 1	I enjoy it; I hate it	I enjoy it	I enjoy it (positive)	
Item 2	I feel bored; I feel interested	I feel interested	I feel bored (negative)	I feel bored (negative)
Item 3	I dislike it; I like it	I liked it	I dislike it (negative)	I dislike it (negative)
Item 4	I find it pleasurable; I find it unpleasurable	I found it pleasurable	I find it pleasurable (positive)	
Item 5	I am very absorbed in this activity; I am not at all absorbed in this activity	I was very absorbed in the activity		
Item 6	It is not fun at all; it is a lot fun	It was a lot fun	It is no fun at all (negative)	It is no fun at all (negative)
Item 7	I find it energizing; I find it tiring		It gives me energy (positive)	
Item 8	It make me depressed; it makes me happy		It makes me sad (negative)	It makes me sad (negative)
Item 9	It is very pleasant; it is very unpleasant	It was very pleasant	It is very pleasant (positive)	
Item 10	I feel good physically while doing it; I feel bad physically while doing it		My body feels good (positive)	
Item 11	It is very invigorating; it is not at all invigorating			
Item 12	I am very frustrated by it; I am not at all frustrated by it		I get something out of it (positive)	
Item 13	It is very gratifying; it is not at all gratifying		It is very exciting (positive)	
Item 14	It is very exhilarating; it is not at all exhilarating		It frustrates me (negative)	It frustrates me (negative)
Item 15	It is not at all stimulation; it is very stimulating		It is not at all interesting (negative)	It is not at all interesting (negative)
Item 16	It gives me a strong sense of accomplishment; it does not give me any sense of accomplishment	I felt as though there was nothing else, I would rather be doing	It gives me a strong feeling of success (positive)	
Item 17	It is very refreshing; it is not at all refreshing		It feels good (positive)	
Item 18	I felt as though I would rather be doing something else; I felt as though there was nothing else		I feel as though I would rather be doing something else (negative)	I feel as though I would rather be doing something else (negative)
Reasons for item deletions	The original scale without deletion	Items seem to tap enjoyment of the activity as well as potential antecedents and consequences of enjoyment	Item 5: the content was not relevant to enjoyment; Item 11: redundant.	Due to the methodological effects behind the positively worded items of the 16-item scale, all positively worded items were deleted.

Note: PACES = physical activity enjoyment scale; the blank cells are the items that were eliminated in the scales/studies.

**Table 2:** Experts’ rating of item relevance, item-level content validity index (I-CVI), and the Kappa designating agreement of relevance (k*) of the 16-item PACES.

Items		Expert 1	Expert 2	Expert 3	Expert 4	Expert 5	Expert 6	Experts in Agreement	I-CVI	P_c_	k*	Evaluation
1	I enjoy it	Y	Y	Y	Y	Y	Y	6	1.00	0.02	1.00	Excellent
2	I feel bored					Y	Y	2	0.33	0.23	0.13	Poor
3	I dislike it	Y	Y				Y	3	0.50	0.31	0.27	Poor
4	I find it pleasurable	Y	Y	Y	Y	Y	Y	6	1.00	0.02	1.00	Excellent
5	It is no fun at all	Y	Y		Y	Y		4	0.67	0.23	0.56	Fair
6	It gives me energy							0	0.00	0.02	−0.02	Poor
7	It makes me depressed							0	0.00	0.02	−0.02	Poor
8	It is very pleasant	Y	Y	Y	Y	Y		5	0.83	0.09	0.82	Excellent
9	My body feels good							0	0.00	0.02	−0.02	Poor
10	I get something out of it							0	0.00	0.02	−0.02	Poor
11	It is very exciting		Y		Y			2	0.33	0.23	0.13	Poor
12	It frustrates me						Y	1	0.17	0.09	0.08	Poor
13	It is not at all interesting		Y					1	0.17	0.09	0.08	Poor
14	It gives me a strong feeling of success							0	0.00	0.02	−0.02	Poor
15	It feels good	Y	Y	Y	Y	Y	Y	6	1.00	0.02	1.00	Excellent
16	I feel as though I would rather be doing something else							0	0.00	0.02	−0.02	Poor

Note: A blank cell implies that the expert’s rating of the item relevance was 1 (not relevant) or 2 (somewhat relevant). “Y” = the expert’s rating of the item relevance was 3 (quite relevant) or 4 (highly relevant).

**Table 3:** Descriptive Statistics and Reliability of the PACES in Study 1.

	*N*	*M* (*SD*)	Minimum Score	Maximum Score	α
Measure 1					
Overall	174	15.75 (3.39)	6	20	0.83
Male	100	15.85 (3.35)	7	20	0.82
Female	74	15.61 (3.46)	6	20	0.85
Measure 2					
Overall	174	15.69 (3.44)	4	20	0.86
Male	98	16.00 (3.54)	4	20	0.87
Female	76	15.29 (3.29)	8	20	0.83

**Table 4:** Factor loadings from exploratory factor analysis of each item in PACES-S.

Item		Factor Loading
1	I enjoy it	0.86
2	I find it pleasurable	0.85
3	It is very pleasant	0.86
4	It feels good	0.79

**Table 5:** Descriptive Statistics and Reliability of the PACES in Study 2.

	*N*	*M* (*SD*)	Minimum Score	Maximum Score	α
Overall	3118	15.99 (3.10)	4	20	0.87
Male	1493	16.25 (3.06)	4	20	0.88
Female	1625	15.75 (3.12)	4	20	0.87

## Data Availability

The datasets generated and analyzed during the current study are not publicly available due to the strict ethical standards required by the Federal Office for the Protection of Data with which study investigators are obliged to comply but are available from the corresponding author on reasonable request.
